# A prediction model of rubber content in the dried root of *Taraxacum kok-saghyz* Rodin based on near-infrared spectroscopy

**DOI:** 10.1186/s13007-024-01183-6

**Published:** 2024-05-26

**Authors:** Runfeng Chen, Qingqing Yan, Tuhanguli Tuoheti, Lin Xu, Qiang Gao, Yan Zhang, Hailong Ren, Lipeng Zheng, Feng Wang, Ya Liu

**Affiliations:** 1 Agricultural College, Xinjiang Agricultural University, Urumqi, 830052 People’s Republic of China; 2 Institute of Crop Germplasm Resource, Xinjiang Academy of Agricultural Sciences, Urumqi, 830091 People’s Republic of China; 3 National Central Asian Characteristic Crop Germplasm Resources Medium-Term Gene Bank (Urumqi), Urumqi, 830091 People’s Republic of China; 4 Crops Research Institute, Guangdong Academy of Agricultural Sciences, Guangdong Provincial Key Laboratory of Crop Genetic Improvement, Guangzhou, 510308 People’s Republic of China; 5 Beijing Linglong Tyre Company Limited, Beijing, 101102 People’s Republic of China; 6 Comprehensive Testing Ground, Xinjiang Academy of Agricultural Sciences, Urumqi, 830052 People’s Republic of China

**Keywords:** Near-infrared spectroscopy, *Taraxacum kok-saghyz*, Natural rubber, Rapid detection, PLS, RF, LightGBM, CNN

## Abstract

**Background:**

*Taraxacum kok-saghyz* Rodin (TKS) is a highly potential source of natural rubber (NR) due to its wide range of suitable planting areas, strong adaptability, and suitability for mechanized planting and harvesting. However, current methods for detecting NR content are relatively cumbersome, necessitating the development of a rapid detection model. This study used near-infrared spectroscopy technology to establish a rapid detection model for NR content in TKS root segments and powder samples. The K445 strain at different growth stages within a year and 129 TKS samples hybridized with dandelion were used to obtain their near-infrared spectral data. The rubber content in the root of the samples was detected using the alkaline boiling method. The Monte Carlo sampling method (MCS) was used to filter abnormal data from the root segments of TKS and powder samples, respectively. The SPXY algorithm was used to divide the training set and validation set in a 3:1 ratio. The original spectrum was preprocessed using moving window smoothing (MWS), standard normalized variate (SNV), multiplicative scatter correction (MSC), and first derivative (FD) algorithms. The competitive adaptive reweighted sampling (CARS) algorithm and the corresponding chemical characteristic bands of NR were used to screen the bands. Partial least squares (PLS), random forest (RF), Lightweight gradient augmentation machine (LightGBM), and convolutional neural network (CNN) algorithms were employed to establish a model using the optimal spectral processing method for three different bands: full band, CARS algorithm, and chemical characteristic bands corresponding to NR. The model with the best predictive performance for high rubber content intervals (rubber content > 15%) was identified.

**Result:**

The results indicated that the optimal rubber content prediction models for TKS root segments and powder samples were MWS–FD CASR–RF and MWS–FD chemical characteristic band RF, respectively. Their respective ${\text{R}}_{{\text{P}}}^{2}$, RMSEP, and RPD_P_ values were 0.951, 0.979, 1.814, 1.133, 4.498, and 6.845. In the high rubber content range, the model based on the LightGBM algorithm had the best prediction performance, with the RMSEP of the root segments and powder samples being 0.752 and 0.918, respectively.

**Conclusions:**

This research indicates that dried TKS root powder samples are more appropriate for constructing a rubber content prediction model than segmented samples, and the predictive capability of root powder samples is superior to that of root segmented samples. Especially in the elevated rubber content range, the model formulated using the LightGBM algorithm has superior predictive performance, which could offer a theoretical basis for the rapid detection technology of TKS content in the future.

## Introduction

*Taraxacum kok-saghyz* Rodin (TKS), commonly referred to as Russian dandelion or turquoise dandelion, is a plant of the Taraxacum genus in the composite family. It is highly tolerant of salt, cold, and drought, making it suitable for planting in a variety of regions. The roots of TKS are capable of synthesizing natural rubber (NR), which is mainly composed of cis-1,4-polyisoprene [1–3]. Reports indicate that the highest NR content in the roots of TKS can reach 27.89%, making it similar in structure and performance to *Hevea brasiliensis*, and even slightly higher in molecular weight than the NR of *H. brasiliensis* [4]. Thus TKS is one of the most promising rubber-producing crops after *H. brasiliensis*. Now, *H. brasiliensis*, the primary source of NR, face challenges such as limited growth areas and susceptibility to South American leaf blight [5, 6], furthermore, political instability and economic fluctuations affect NR pricing and availability [7]. The global NR market, with was valued at $24 billion in 2016, was expected to grow to 16.5 million tons by 2023 and $68.5 billion by 2026 [8]. Therefore, there is an urgent demand to develop a secondary source of rubber and the industrialization of TKS is a pressing requirement. Currently, the TKS industry is still in its nascent phase, with the NR content of artificially cultivated TKS is typically being low. Consequently, breeding initiatives is crucial for advancement of the current TKS industry. This breeding initiatives have a lot of necessitates on the content of NR content testing, however, existing methods for detecting NR content are often time-consuming, labor-intensive, or costly. Such as the alkali boiling [9], gravimetry [10], Soxhlet extraction [11–13], Nuclear magnetic resonance spectroscopy (NMR) [14, 15], accelerated solvent extraction (ASE) [16, 17], and pyrolysis gas chromatography (Py-GC) [18], etc. Therefore, there is an urgent need for a fast, accurate, and low-cost method to detect the NR content of TKS.

Near infrared spectroscopy (NIR) has experienced rapid development in recent years [19]. This technology, an organic integration of spectral measurement, computer technology, and foundational measurement techniques, offers unique advantages such as non-destructive testing and low analysis costs. It records the overtones and combination tones of the fundamental frequency vibrations of chemical bonds such as C–H, O–H, N–H, and other hydrogen-containing groups in a molecule for qualitative or quantitative analysis [20], and has been extensively applied in sectors like medicine, food, and agricultural production [21]. Studies have successfully established prediction models for the NR content in *Parthenium hysterophorus* L using NIR technology. Suchat et al. [22] developed a PLS quantitative model for NR content in *P. hysterophorus* L using standard normalized variate (SNV), de-trending (DT), and derivative-processed spectra, achieving an R^2^ of 0.96. Taurines et al. [23] utilized SNV and derivative-processed spectra to establish a PLS model for NR content in *P. hysterophorus* L powder samples, with a predictive set R^2^ of 0.95. Luo et al. [24] attempted preprocessing with smoothing, DT, SNV, and derivatives, creating a PLS model for NR content in *P. hysterophorus* L with a cross-validation set R^2^ of 0.79. García-Martínez et al. [25] preprocessed the spectra with smoothing, SNV, DT, and derivatives to establish a PLS model for NR content in *P. hysterophorus* L, achieving a cross-validation set R^2^ of 0.9 and an relative percentage deviation (RPD) of 2.65. These findings confirm that preprocessing methods like smoothing, SNV, and derivatives can effectively remove some environmental errors in the spectra and enhance spectral features related to NR content. In 2022, Chen et al. [26] discovered that the NIR spectral range of the TKS roots contains characteristic bands with higher resolution of NR and successfully established a PLS prediction model for the NR content in fresh TKS roots, with a predictive set R^2^ of 0.97. However, there are no reports in the literature on NR content prediction models for dry TKS roots.

This study aims to collect spectral data of TKS root samples treated with two different methods, namely root segment and powder, within the range of 850–2500 nm. By combining with stoichiometric methods and utilizing preprocessing techniques such as moving window smoothing (MWS), SNV, multiplicative scatter correction (MSC), and first derivative (FD), the study establishes a near-infrared spectral quantitative model suitable for rapid determination of NR content in TKS dry roots. This approach includes smoothing, SNV, and derivative processing, which have been previously employed in rubber content prediction models [22, 24], and MSC, a method similar to SNV, frequently used in the establishment of spectral quantitative models [27]. Current rubber content prediction models are predominantly linear, with PLS being the sole modeling algorithm applied in previous studies [22–26]. Therefore, in addition to PLS, this study incorporates three nonlinear modeling algorithms commonly used in quantitative model establishment: random forest (RF), lightweight gradient augmentation machine learning (LightGBM), and convolutional neural network (CNN), for comparison. The objective is to identify a more suitable algorithm for predicting rubber content in dry TKS roots, thereby providing technical support for the rapid and accurate determination of NR content in TKS and advancing the breeding work of TKS.

## Materials and methods

### Test materials

**Fig. 1:**
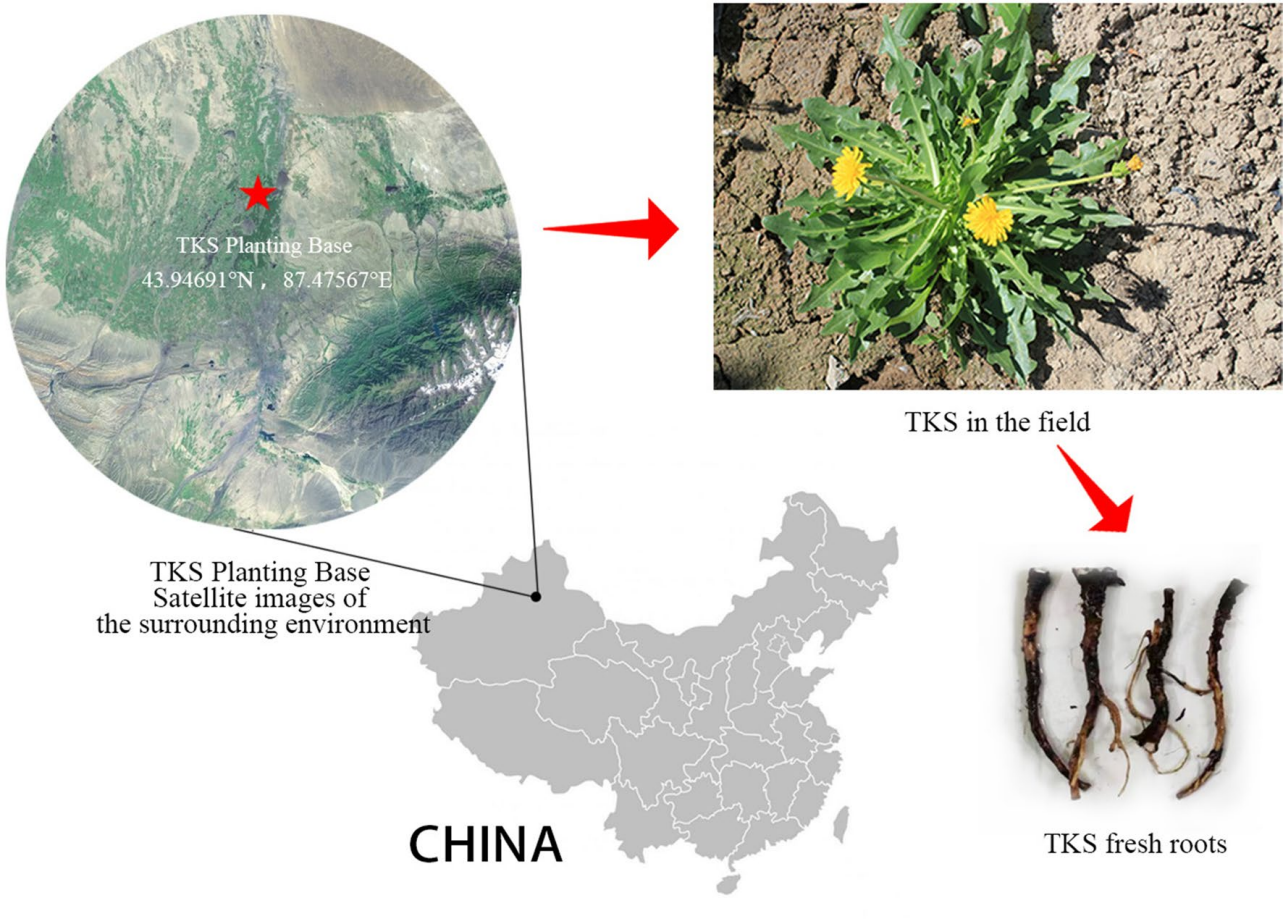
Environmental satellite image around TKS Planting Base, along with TKS samples

This experiment utilized 129 TKS samples of the K445 strain, some of which were hybridized with other dandelion plants and harvested at various stages of growth throughout the year in 2023. All of the test samples were obtained from the TKS Planting Base of the Xinjiang Academy of Agricultural Sciences Comprehensive Testing Ground in Urumqi, Xinjiang, China, situated at 43.94691°N and 87.47567°E (Fig. 1). Upon collection, the TKS samples were processed within 48 h. The above-ground parts were removed using scissors, leaving only the roots, which were then cleaned to remove soil and other impurities. Subsequently, the roots were dried in an oven at 80 °C until completely moisture-free and stored individually in brown paper bags for preservation.

### Collection of spectral information

**Fig. 2:**
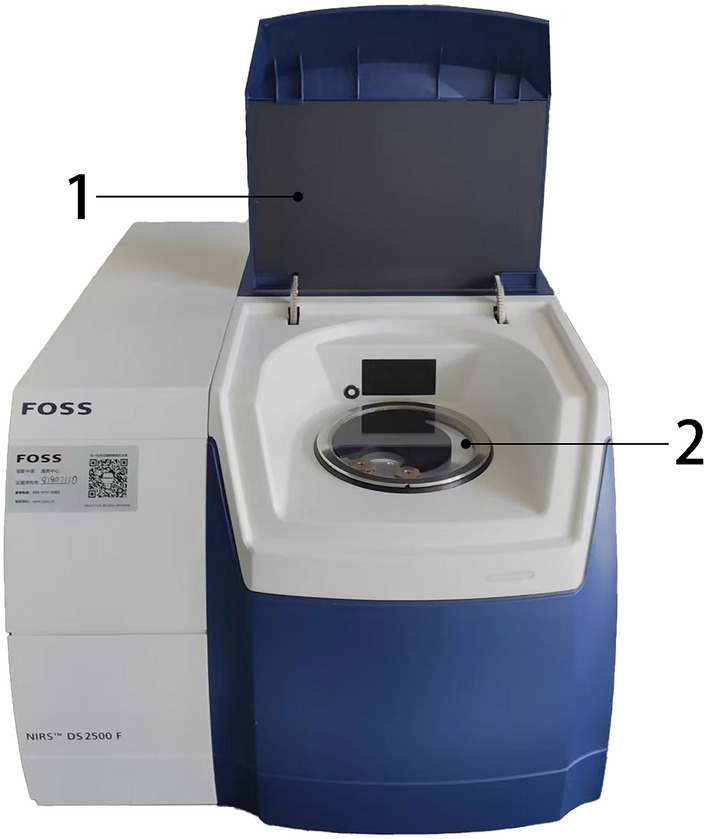
FOSS NIRS™ DS2500F SR diffuse reflection near-infrared spectrometer (1 sample test bin covers 2 sample diffuse reflection test bin)

The FOSS NIRS™ DS2500F SR (Fig. 2) spectrometer from Flowserve Company was utilized to collect diffuse reflectance spectra of a sample. The spectral collection range was from 850 to 2500 nm, with a spectral resolution of 0.5 nm. To examine the effects of different forms of TKS roots on the performance of spectral collection and prediction models, two sample preparation methods were used: cutting and grinding. Initially, each sample was cut into small sections with a length of 5 mm and a diameter of less than 5 mm. These root sections were then put through spectral collection. Afterward, the samples were soaked in liquid nitrogen and frozen for 1 min to embrittle them, followed by grinding for 3 min using the JXFSTPRP-CLN-48 frozen grinding machine from NetEason. The particle size of the ground powder was smaller than 0.097 mm (capable of passing through a 180 mesh sieve). The powder samples were then sent to a spectrometer for spectral collection. Altogether, 129 samples were collected for root segment and powder state spectra. To reduce errors caused by particle size factors, the sample inversion was repeated three times during spectrum collection and the average spectrum was taken. Before spectral collection, the instrument was preheated for at least an hour, and the spectra were scanned seven times each time, with a total of 3300 spectral points collected each time. After collection, the spectra were simplified and adjusted to spectral data with a wavelength resolution of 2 nm, with each sample spectrum consisting of 825 wavelengths.

### Determination of NR content

This experiment employed the alkali boiling method to measure the NR content of TKS roots. This method has an impurity purity of 93.77% [9], thus it is likely to yield slightly higher results; however, this error is unlikely to significantly affect the breeding results.

To begin, the roots of TKS were dried and cut into small pieces of 0.5 cm. 0.5 g of the sample was placed in a glass test tube and 10 ml of 3% sodium hydroxide solution was added. The sample was then boiled in a water bath for 2 h. After the boiling bath, the sample was rinsed 5–8 times with distilled water and 15 ml of distilled water was added for 5–10 min. The sample was then placed in a mortar, pressed, and rinsed to separate the NR from the roots. The cleaning solution was checked for any turbidity and the rubber block was removed and squeezed dry. The sample was then placed in a 1% hydrochloric acid solution, neutralized for 5–10 min. The surface alkaline substances generated by the reaction with sodium hydroxide solution were removed to stabilize the pH of the samples, and cleaned and dehydrated with 96% alcohol for 20–20 min, to facilitate easier drying, and phenomenon of rubber turning black can also be significantly alleviated [28]. Finally, the sample was dried in an oven at 80 ℃ and the weight was recorded.

**Fig. 3:**
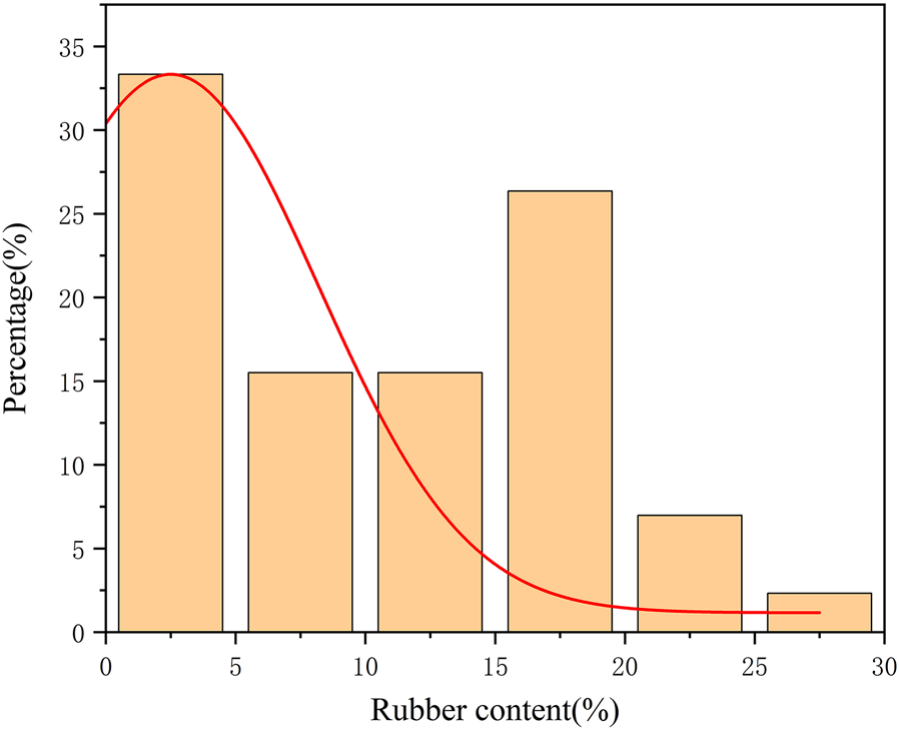
Distribution of frequency of NR content in the sample set

As seen in Table 1 and Fig. 3, the NR content of the sample ranged from 0 to 28.7%, with an average value of 10.49%. The presence of hybrid plants in the sample caused a large number of samples to have low content (NR content range of 0–5%).

**Table 1:** Statistical table of NR content in the sample set

Sample number	NR content (%)					
	Maximum	Minimum	Median	Mean	Standard deviation	Coefficient of variation (CV)
129	28.70	0.00	10.64	10.49	7.76	73.97%

### Spectral data processing methods

This experiment used MATLAB 2019b to preprocess spectral data to improve the predictive performance of the model. Monte Carlo sampling (MCS) was used to remove any abnormal data from the sample set. This was due to the differences in the morphology, size, and particle size of the cut root segments and ground powder samples, which caused a change in the optical path during the diffuse reflection process, resulting in poor spectral repeatability. To reduce spectral errors caused by environmental factors, MWS, SNV, MSC, and FD processing were applied to the spectral data. MWS, which requires the selection of a window with a predefined size, moves across the spectrum and replaces the measured values at each wavelength point with the calculated average at the center wavelength of the window [29]. In this study, the spectral window size for MWS filtering was set to five spectral segments. MWS can reduce some noise in the spectrum, thereby reducing the impact of environmental errors on the spectrum [30]. SNV and MSC are similar in algorithm; both standardize the spectral data. The main difference between them is that SNV uses specific spectral data to normalize each spectrum, while MSC uses data from the entire dataset to standardize the spectrum [31]. SNV can reduce interference caused by physical differences in samples [32, 33], and MSC can eliminate wavelength shifts caused by sample scattering [34]. Derivative is a commonly used spectral preprocessing method in the establishment of rubber content prediction models [22–26], FD algorithm has the advantage of eliminating baseline drift and stacking effects, improving spectral resolution, and effectively removing interference from constant baselines and backgrounds [35].

The NR in TKS is a biopolymer composed of isoprene units (C5H8)_n_ in a 1,4-cis configuration[36], which possess a few hydrogen functional group bands in the near-infrared spectrum. However, due to environmental and other factors, there exist some noise bands in the near-infrared spectrum which can hinder the predictive performance of the model. To address this issue, the competitive adaptive reweighted sampling (CARS) method [37] and the previously discovered characteristic bands of NR of TKS [26] were employed to screen the spectra and reduce the dimensionality of the data, thus reducing the computational complexity and partial noise of the model and minimizing the risk of overfitting. The constrained algorithm for regression variable selection (CARS) is a method that combines MCS with the regression coefficients of partial least squares (PLS) model for feature variable selection, mimicking the principle of “survival of the fittest” from Darwin’s theory [37]. In the CARS algorithm, each iteration retains points with higher absolute weight of regression coefficients in the PLS model through adaptive reweighted sampling (ARS), discarding those with lower weights. A PLS model is then built based on the new subset. After multiple iterations, the wavelengths in the subset with the smallest root mean square error of cross-validation (RMSECV) are selected as characteristic wavelengths. CARS is commonly used as a spectral feature wavelength selection method for the establishment of spectral prediction models [27]. However, this algorithm has not yet been applied to the selection of rubber wavelength characteristic wavelengths. This experiment will compare the wavelengths selected by the CARS algorithm with the characteristic wavelengths of NR discovered by previous researchers to identify a more suitable wavelength selection method for the establishment of prediction models for the content of NR in TKS.

### Model building method

This study utilized Python 3.10 to create a model and employed four linear and nonlinear methods to forecast the NR content of TKS, including PLS, RF, lightweight gradient boosting machine (LightGBM), and CNNs.

PLS is a type of multiple linear regression model that amalgamates the benefits of three analysis techniques: principal component analysis, canonical correlation analysis, and multiple linear regression analysis. It resolves the issue of having more samples than variables in multiple linear regression models and is effective when the variables are highly linearly correlated. It has been used to construct an NR content prediction model for TKS fresh roots [26], displaying impressive predictive performance.

RF [38] and LightGBM [39] are both isomorphic ensemble learning algorithms based on decision trees. RF is a parallel structure utilizing bagging, where each decision tree is independent and the final prediction result is determined through voting on the constructed decision trees. LightGBM, proposed by Ke et al. [39] from Microsoft Research Institute in 2017, is a serial structure based on boosting. It is more efficient in terms of training, accuracy, and memory usage than other boosting frameworks such as GBRT and XGBoost due to the introduction of the gradient based one side sampling (GOSS) algorithm and exclusive feature binding (EFB) technology. GOSS reduces the number of data instances with small gradients, while EFB merges multiple mutually exclusive features into one feature, thus achieving dimensionality reduction. In this study, when establishing the RF model, we set the number of decision trees (n_estimators) to 200 and the maximum depth of the tree (max_depth) to the default value Noen, which allows the tree to grow naturally. When establishing the LightGBM model, we set the learning rate (learning_rate) to the default value of 0.1, the maximum depth of the tree (max_depth) to −1, which allows the model to automatically determine the maximum depth of the tree, and the maximum number of leaves (num_leaves) to 30.

CNNs are a widely utilized technique in data analysis and are a prominent example of deep learning technology [40]. They are capable of analyzing one-dimensional data [41–43] and are composed of convolutional layers, pooling layers, and fully connected layers. Convolutional layers extract local feature information from the input data by applying convolutional kernels to the spectral data, and multiple convolutional layers can be stacked to deepen the network structure and improve the model’s feature representation capabilities. The pooling layer simplifies the model by reducing the dimensionality of the input features, while the fully connected layer connects the output of the previous layer to the desired target output, thus establishing a relationship between the extracted feature information and the target output.

**Fig. 4:**
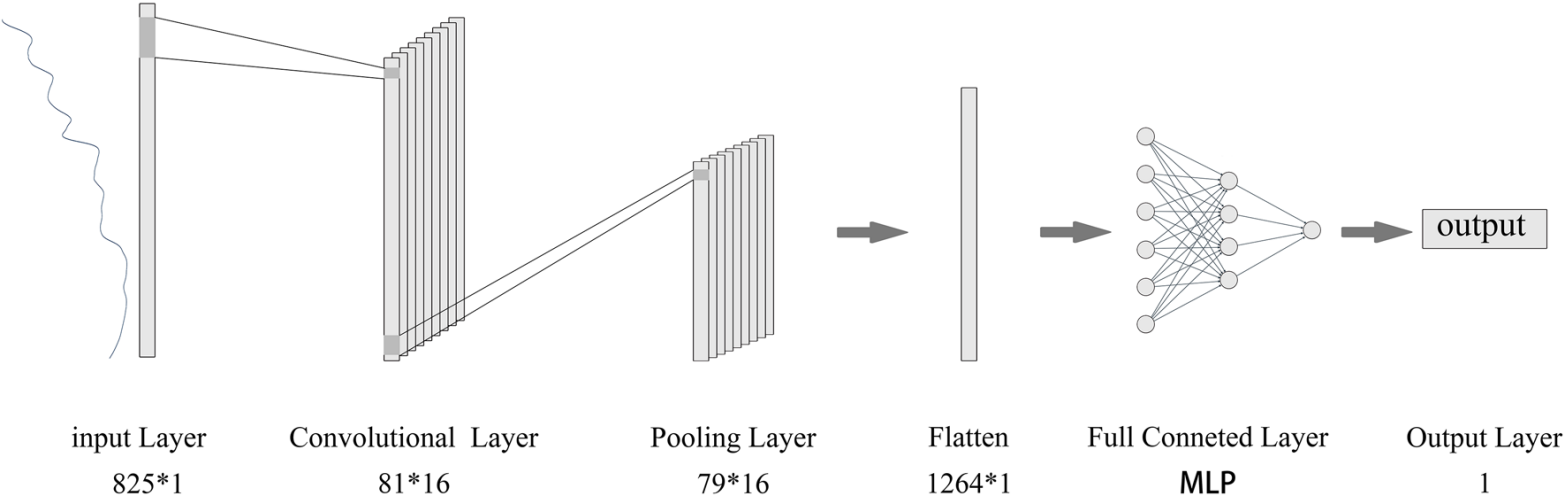
Structure diagram of NR content model of TKS based on 1-D CNN

This study establishes a CNN model based on the PyTorch framework. Since CNN has not yet been utilized for the development of prediction models for NR content, there is a lack of reference for the optimal setting of hyperparameters. Therefore, this experiment references the parameters set in the 1D-CNN regression model of other plants [44, 45] and makes adjustments to design a 1D-CNN model structure suitable for the experimental data. The basic structure of the model is illustrated in Fig. 4. The model comprises one input layer, three hidden layers (one convolutional layer, one pooling layer, and one fully connected layer), and one output layer. The convolutional layer has a kernel size of 20 * 1, a stride of 10, and 16 kernels, which are used to extract various local features from the input data and obtain local abstract feature maps. The output data from the convolutional layer is passed to the pooling layer, where maximum pooling is applied with a kernel size of 3 * 1 and a stride of 1, further refining the features and reducing the length of the output feature vectors from the convolutional layer. The fully connected layer utilizes an MLP model, with the pooling layer’s output data being input into the fully connected layer, and the output data being the predicted value of NR. The model employs stochastic gradient descent (SGD) as the optimizer, with mean squared error (MSE) serving as the loss function. The learning rate for training the network model is set to 0.01, with 20,000 learning epochs, and ReLU is used as the activation function for all hidden layers.

### Model evaluation method

The coefficient of determination (R^2^), root mean square error (RMSE), and RPD are utilized to evaluate the predictive performance of the model. A higher R^2^ and lower RMSE indicate better predictive performance [46]. In the detection of complex samples, it is typically accepted that an RPD > 2 is sufficient for initial screening, and an RPD > 3 indicates good predictive performance [47]. Ultimately, the evaluation parameters of the prediction model established by the full band and processed spectra are compared to identify the optimal method for model establishment. The calculation equations for R^2^, RMSE, and RPD are as follows: Eq. (1), (2), and (3).

1 $${\text{R}}^{2} = 1 - \frac{{\sum\nolimits_{{{\text{i}} = 1}}^{{\text{n}}} {\left( {{\text{y}}_{{\text{i}}} - \widehat{{\text{y}}}_{{\text{i}}} } \right)^{2} } }}{{\sum\nolimits_{{{\text{i}} = 1}}^{{\text{n}}} {\left( {{\text{y}}_{{\text{i}}} - \overline{{\text{y}}}_{{\text{i}}} } \right)^{2} } }}$$

2 $${\text{RMSE}} = \sqrt {\frac{{\sum\nolimits_{{{\text{i}} = 1}}^{{\text{n}}} {\left( {{\text{y}}_{{\text{i}}} - \widehat{{\text{y}}}_{{\text{i}}} } \right)^{2} } }}{{\text{n}}}}$$

3 $${\text{RPD}} = \frac{{\sum\nolimits_{{{\text{i}} = 1}}^{{\text{n}}} {\left( {{\text{y}}_{{\text{i}}} - \overline{{\text{y}}}_{{\text{i}}} } \right)^{2} } }}{{{\text{RMSE}}}}$$

In the formula, is the true value of sample i, is the predicted value of sample i, and is the average value of sample i.

## Results

### Abnormal data deletion

**Fig. 5:**
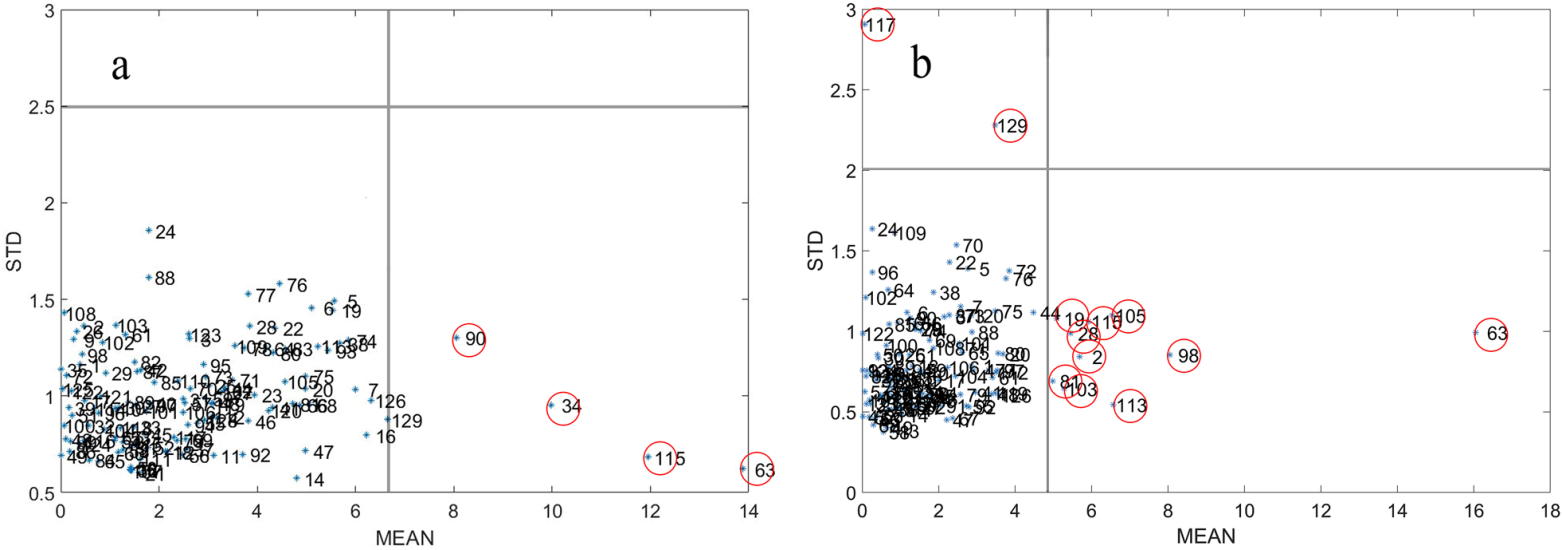
Monte Carlo outlier detection diagram of TKS root segment (**a**) and powder (**b**) sample

**Fig. 6:**
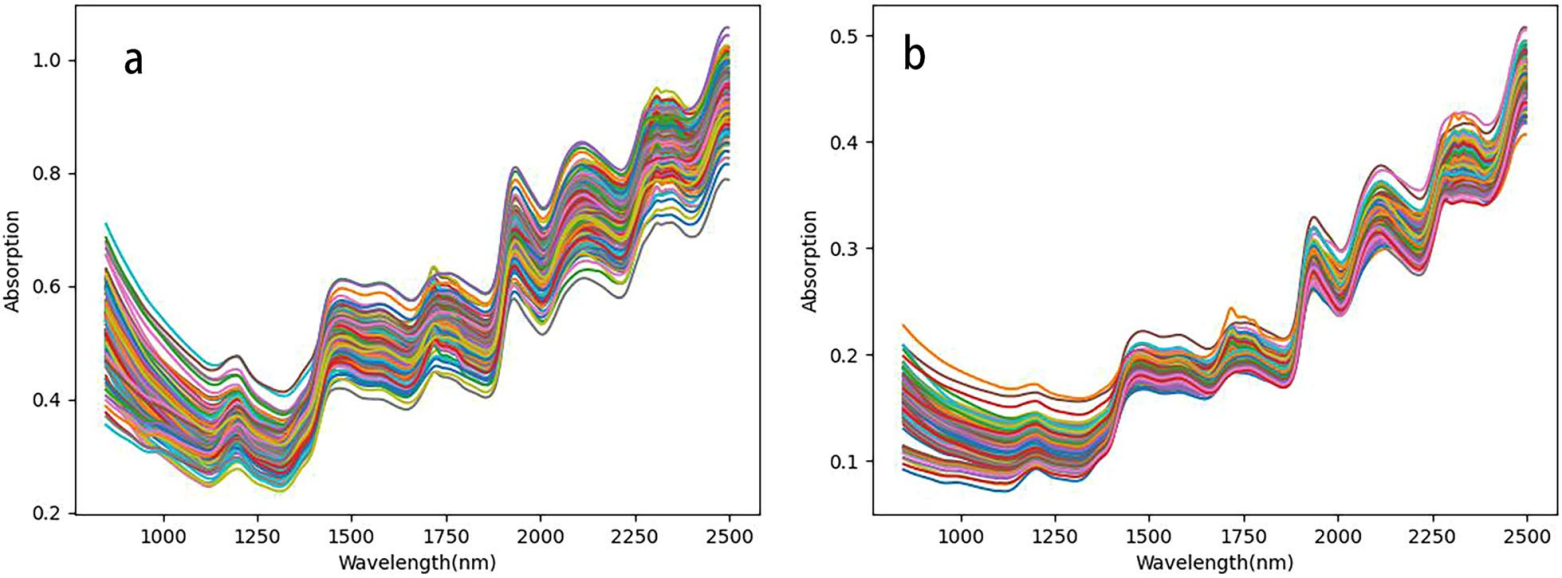
Original spectra of TKS roots segment (**a**) and powder (**b**) after removal of abnormal data

MCS was employed to calculate the mean prediction error (MEAN) and standard deviation of prediction error (STD) for 129 TKS root segments and powder spectral sets. These two values were used to construct a right-angle coordinate system and plot a scatter plot [48]. The thresholds for root segment samples and powder samples were MEAN = 6.72%, STD = 2.49 and MEAN = 4.87%, STD = 2.00, respectively. As shown in Fig. 5, there were 4 root segment (Fig.5a) sample data and 12 powder (Fig.5b) sample data located outside the threshold segmentation line. PLS were applied to establish a prediction model for the data before and after removal, and cross validation was conducted. The results showed that the ${\text{R}}_{{{\text{CV}}}}^{2}$ and RMSECV of the TKS root segment and powder PLS models increased after data removal (Table 2), indicating that there were indeed anomalies in the data. Consequently, these data were removed, resulting in 125 root segment sample datasets and 117 powder sample datasets. Fig. 6 shows the original near-infrared spectrum after removing abnormal data.

**Table 2:** Prediction effect of the PLS model before and after sample removal by MCS method

Sample status	Monte Carlo culls numbers	Model evaluation parameter				Principal component number
		${\text{R}}_{{\text{c}}}^{2}$	RMSEC	${\text{R}}_{{{\text{CV}}}}^{2}$	RMSECV	
Root segment	0	0.835	3.147	0.724	3.605	8
	4	0.885	2.557	0.814	2.867	8
Root powder	0	0.897	2.486	0.843	2.749	8
	12	0.960	1.533	0.916	1.893	8

### Division of sample set

The SPXY algorithm [49] was employed to partition the training set and validation set. This method is an improved version of the KS (Kolmogorov Smirnov) algorithm, as it calculates the Euclidean distance of the x-vector direction (i.e. spectral data) as well as the y-vector direction (i.e. the measured values of the samples) of different samples, and combines them through regularization for a more thorough assessment and separation of the dataset. After removing abnormal data from the root segment and powder samples, the training and validation sets were divided into 125 and 117 datasets, respectively, in a 3:1 ratio. As shown in Table 3, the root segment sample dataset was divided into 94 training set data and 31 validation set data, while the powder sample was divided into 88 training set data and 29 validation set data.

**Table 3:** Statistical data of NR content of TKS in sample set divided by SPXY method

Sample status	Sample set	Sample size	NR content (%)			
			Maximum	Minimum	Mean	Standard deviation
Root segment	Training set	94	28.70	0	10.67	7.47
	Validation set	31	20.77	0	9.30	7.83
Root powder	Training set	88	28.70	0	10.77	7.65
	Validation set	29	19.43	0	8.25	7.54

### Spectral data preprocessing

This experiment employed four distinct spectral preprocessing techniques. Following data preprocessing, the SPXY algorithm was used to divide the training and validation sets. Using the training set data of root segments and powder samples, a PLS, RF, LightGBM, and CNN model were all established to predict the NR content in TKS roots. The validation set was used to calculate the evaluation parameters of the model, the results of which are presented in Tables 4 and 5. The preprocessed spectral data improved the predictive performance in comparison to the unprocessed data. RPD_P_ was used to assess the predictive performance of the model, with the MWS-FD-RF model displaying the best results for root segment samples, with an RPD_P_ of 4.111; the best model for powder samples was the MWS-FD-CNN model, with an RPD_P_ of 5.717.

**Table 4:** Evaluation parameters of different models of NR content for the raw NIR spectral data of the TKS roots segment and different pre-treated spectral data

Modeling method	Spectral processing method	Training set		Validation set		RPD_P_
		${\text{R}}_{{\text{c}}}^{2}$	RMSEC	${\text{R}}_{{\text{P}}}^{2}$	RMSEP	
PLS	None	0.881	2.562	0.868	2.796	2.757
	MWS	0.898	2.375	0.887	2.588	2.978
	MWS-SNV	0.882	2.514	0.904	2.320	3.230
	MWS-MSC	0.880	2.533	0.886	2.538	2.958
	MWS-FD	0.896	2.291	0.929	2.192	3.757
RF	None	0.932	1.938	0.732	3.993	1.930
	MWS	0.935	1.896	0.747	3.874	1.989
	MWS-SNV	0.965	1.360	0.908	2.272	3.298
	MWS-MSC	0.966	1.352	0.919	2.139	3.510
	MWS-FD	0.971	1.199	0.941	2.003	4.111
LightGBM	None	0.876	2.620	0.806	3.391	2.273
	MWS	0.880	2.573	0.809	3.368	2.288
	MWS-SNV	0.975	1.152	0.920	2.124	3.529
	MWS-MSC	0.977	1.112	0.926	2.035	3.688
	MWS-FD	0.987	0.796	0.918	2.357	3.494
CNN	None	0.935	1.960	0.911	2.348	3.283
	MWS	0.931	1.973	0.915	2.293	3.362
	MWS-SNV	0.938	1.833	0.938	2.031	3.690
	MWS-MSC	0.939	1.804	0.913	2.227	3.370
	MWS-FD	0.975	1.123	0.938	2.059	4.000

**Table 5:** Evaluation parameters of different models of NR content based on raw NIR spectral data of TKS roots powder and different pretreatment spectral data

Modeling method	Spectral processing method	Training set		Validation set		RPD_P_
		${\text{R}}_{{\text{c}}}^{2}$	RMSEC	${\text{R}}_{{\text{P}}}^{2}$	RMSEP	
PLS	None	0.958	1.570	0.960	1.492	4.970
	MWS	0.957	1.571	0.959	1.493	4.966
	MWS–SNV	0.959	1.524	0.964	1.425	5.307
	MWS–MSC	0.955	1.592	0.960	1.520	4.975
	MWS–FD	0.968	1.345	0.956	1.696	4.776
RF	None	0.943	1.823	0.815	3.194	2.322
	MWS	0.945	1.784	0.800	3.316	2.236
	MWS–SNV	0.981	1.041	0.956	1.580	4.787
	MWS–MSC	0.979	1.105	0.956	1.588	4.761
	MWS–FD	0.989	0.781	0.966	1.484	5.461
LightGBM	None	0.923	2.109	0.745	3.744	1.981
	MWS	0.923	2.111	0.770	3.560	2.083
	MWS–SNV	0.985	0.910	0.964	1.427	5.297
	MWS–MSC	0.985	0.910	0.946	1.761	4.294
	MWS–FD	0.990	0.763	0.960	1.630	4.970
CNN	None	0.975	1.246	0.968	1.381	5.371
	MWS	0.974	1.250	0.966	1.397	5.308
	MWS–SNV	0.982	0.999	0.969	1.406	5.376
	MWS–MSC	0.981	1.057	0.971	1.384	5.462
	MWS–FD	0.989	0.798	0.970	1.417	5.717

### Feature wavelength screening

#### Competitive adaptive Reweighted sampling method (CARS) screened the wavelength

**Fig. 7:**
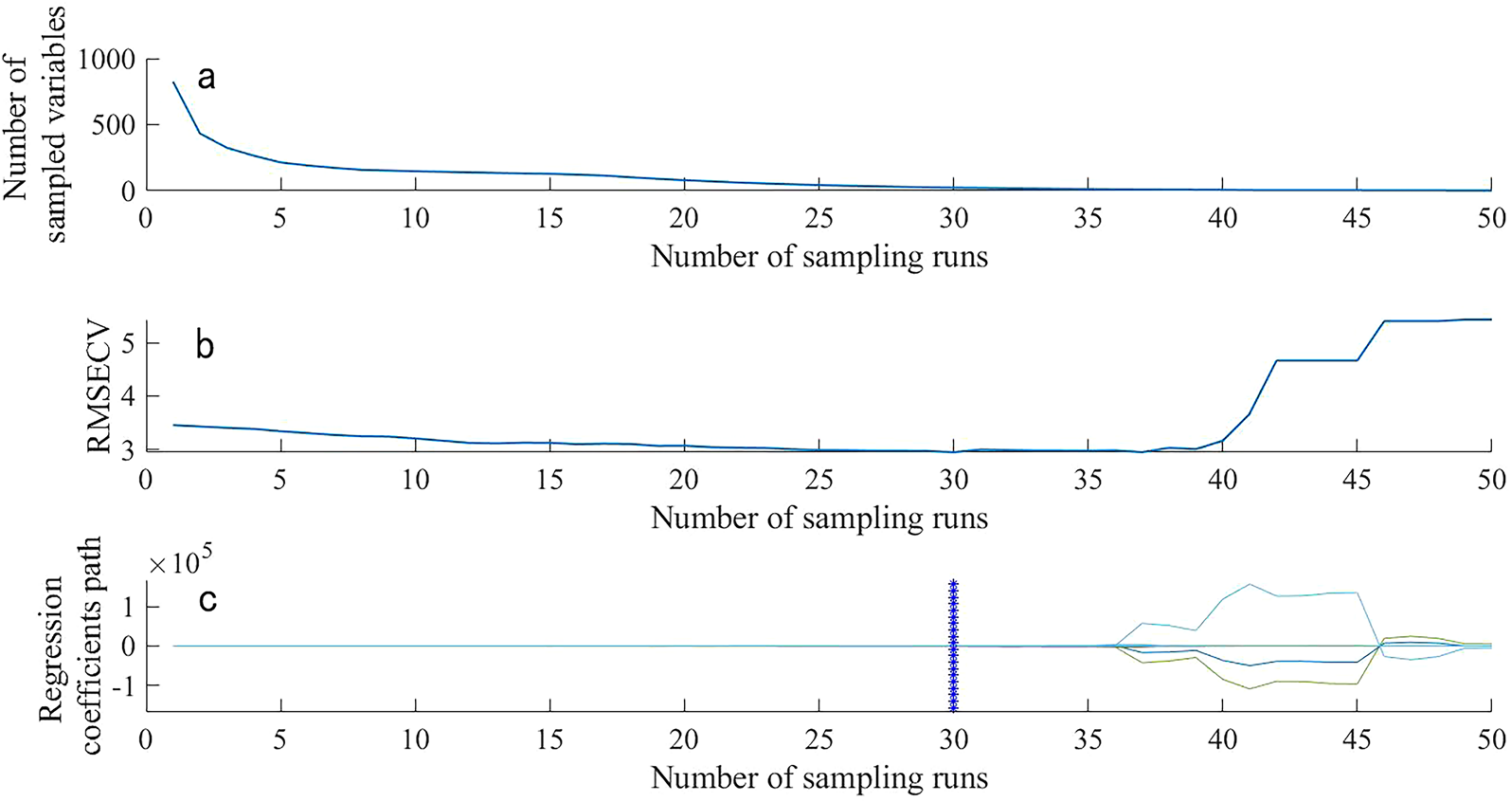
Process of CARS screening the spectral characteristic wavelength of TKS roots segment samples

**Fig. 8:**
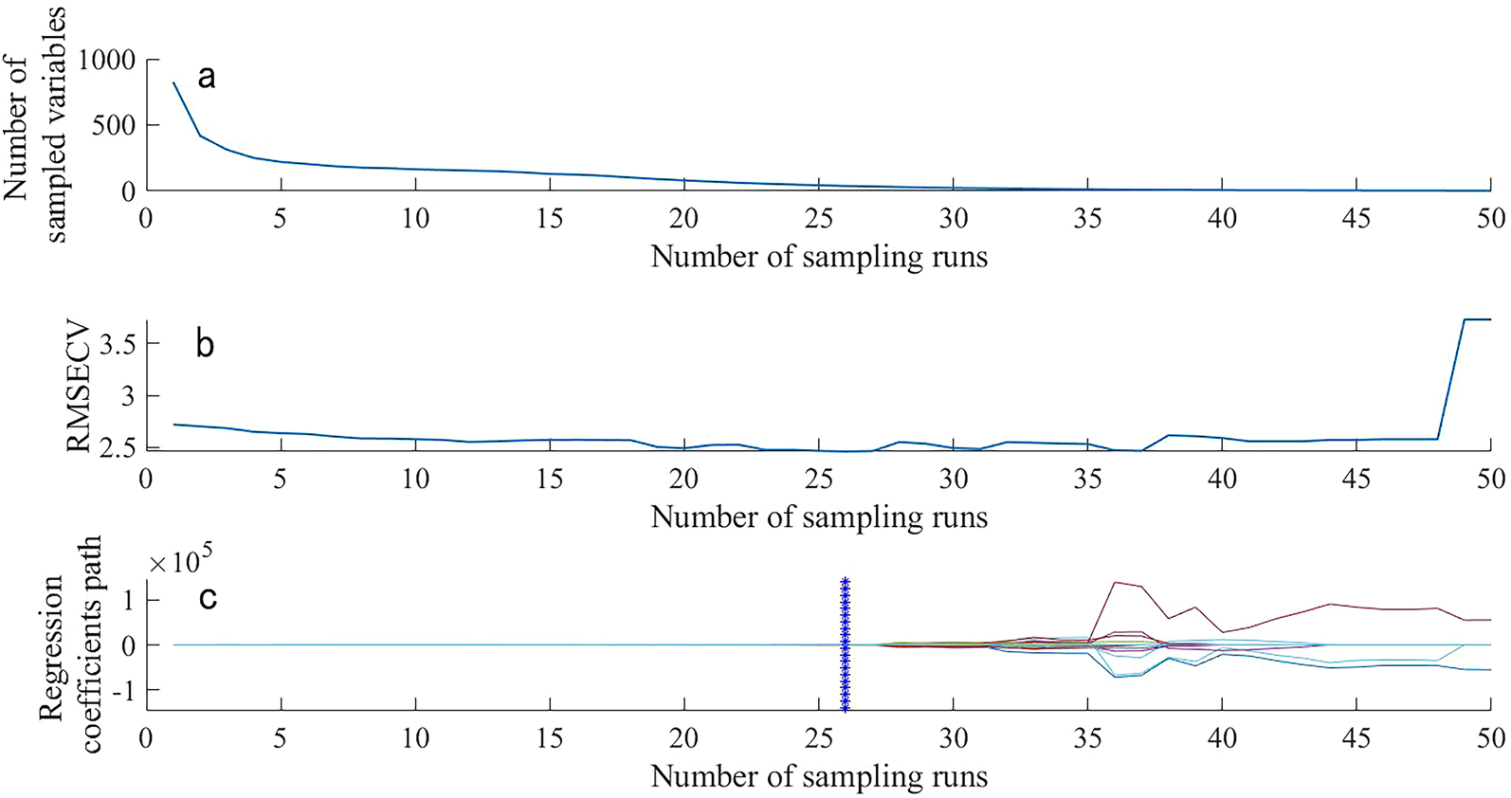
Process of selecting the characteristic wavelength of TKS roots powder by CARS

Figure. 7 and 8 represent the process of extracting NR characteristic wavebands from the near-infrared spectra of rubber tree root segments and powder, respectively, using the CARS algorithm with 50 Monte Carlo sampling iterations. From Fig.7a, it can be observed that with the increase in sampling iterations, the wavelengths with low contribution rates to the prediction performance of the rubber tree root segment spectral model are continuously being eliminated. Figure.7b shows that when the number of iterations reaches 30, the root mean square error of cross-validation set (RMSECV) reaches the lowest point and then starts to rise, indicating that further sampling would eliminate the characteristic wavelengths of NR. Figure.7c depicts the relationship between the regression coefficients of wavelength variables and the number of sampling iterations, with the best number of iterations marked by a vertical line composed of "*" at 30 iterations, resulting in the selection of 26 characteristic NR wavelengths, accounting for approximately 3.15% of the total wavelength. Similarly, Fig.8a shows that with the increase in sampling iterations, low-contributing wavelengths are continuously being reduced. From Fig.8b, it can be seen that the RMSECV of the near-infrared spectrum of rubber tree root powder reaches the lowest point at 27 iterations. The best number of iterations is indicated by a vertical line composed of "*" in Fig. 8c, resulting in the selection of 34 characteristic wavelengths, which account for 3.6% of the total wavelength.

#### Rubber chemical characteristic bands in TKS

**Fig. 9:**
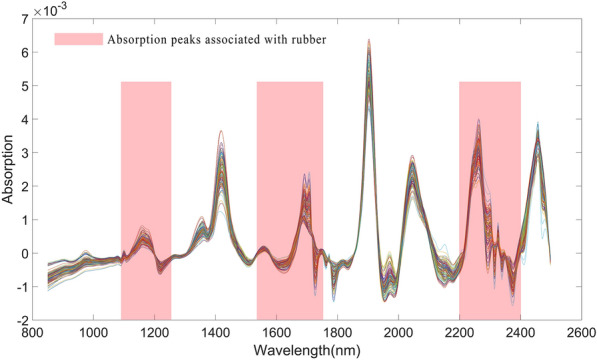
NR characteristic wavelength interval of near infrared spectrum of TKS root after FD

Polyisoprene, the main component of NR, has characteristic wavelengths in the near-infrared spectrum of TKS roots, which range from 1100–1250 nm, 1550–1760 nm, and 2100–2400 nm[26], and account for 40% of the total wavelength (Fig. 9). This band contains the characteristic wavelengths of the –CH, –CH_2_, and –CH_3_ functional groups in cis polyisoprene [50, 51], which can enhance the accuracy of model prediction.

### Optimal model screening

#### Evaluation of prediction performance of different models

**Fig. 10:**
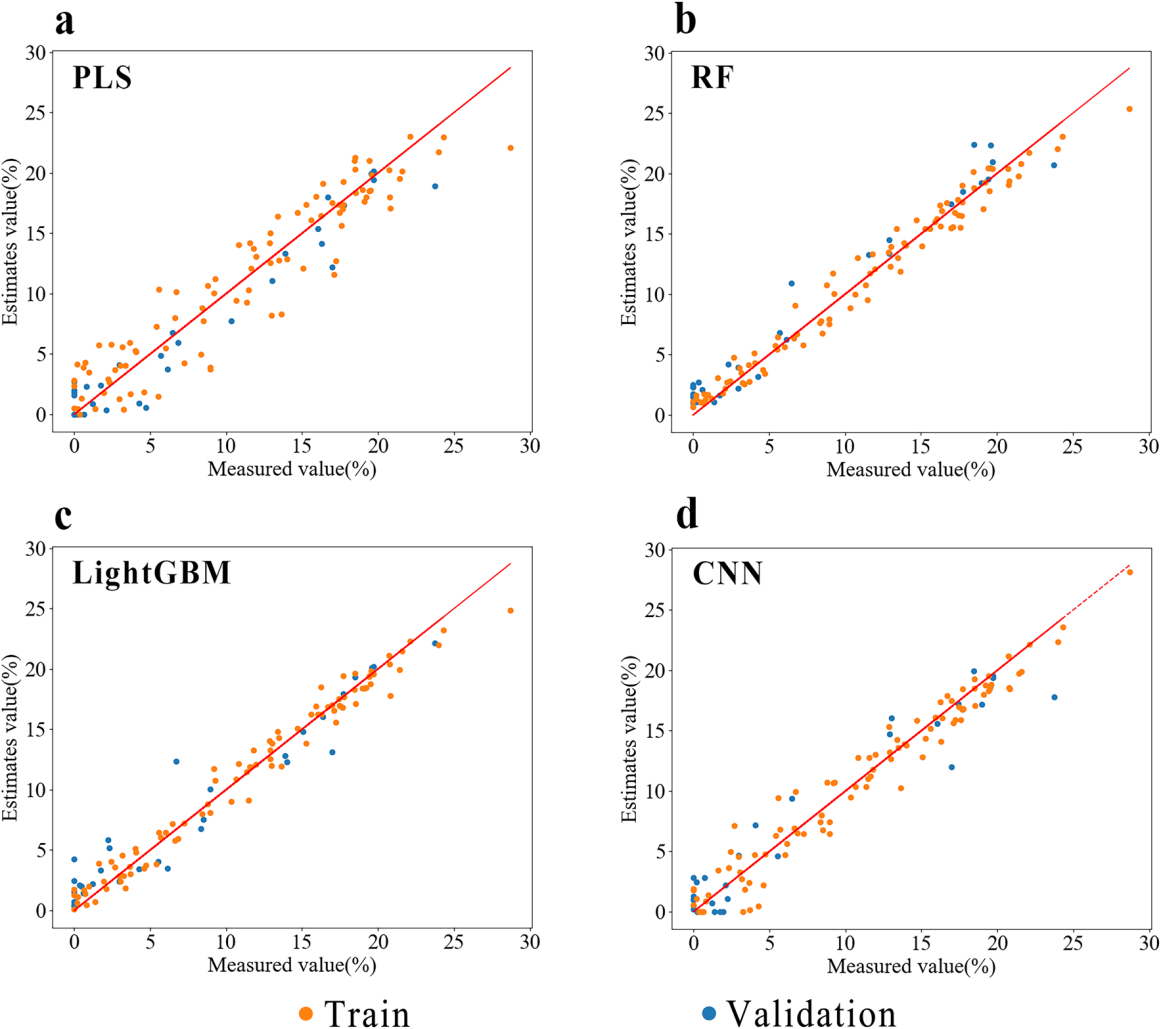
Optimal model of PLS (**a**), RF (**b**), LightGBM (**c**) and CNN (**d**). Scatter plot of measured and predicted NR content in TKS roots segment samples

**Fig. 11:**
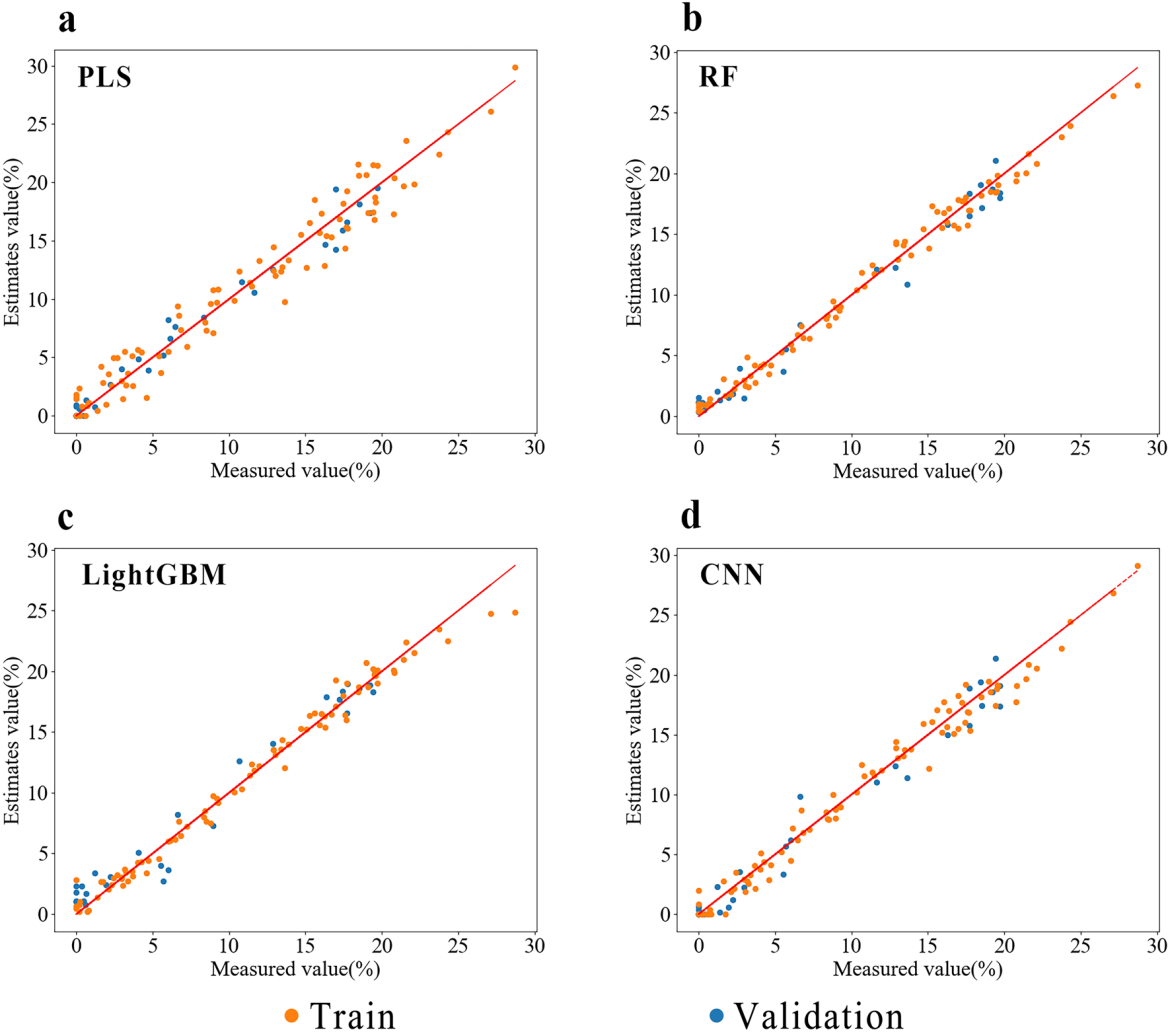
Optimal model of PLS (**a**), RF (**b**), LightGBM (**c**) and CNN (**d**). Scatter plot of measured and predicted NR content in TKS root powder samples

The optimal preprocessing scheme for each modeling method was used separately, and a model was established after band screening to predict the data in the validation set. The evaluation parameter results of different models are presented in Tables 6 and 7, and the scatter plots of predicted and measured values of different models are shown in Figs. 10 and 11. After wavelength screening, the upper limit of the model’s prediction performance was improved. Among them, the MWS-FD-CASR-RF model had the best prediction performance for TKS root segment samples, with an RPD_P_ of 4.498 from 4.111. The MWS-FD-Chemical Characteristic Band-RF model had the best prediction performance for powder samples, with an RPD_P_ of 5.461 to 6.845.

**Table 6:** Effects of different wavelength screening methods on the performance of TKS roots segment sample prediction model

Modeling method	Optimal spectral processing	Wavelength screening method	Training set		Validation set		RPD_P_
			${\text{R}}_{{\text{c}}}^{2}$	RMSEC	${\text{R}}_{{\text{P}}}^{2}$	RMSEP	
PLS	MWS-SNV	Full band	0.896	2.291	0.929	2.192	3.757
		CARS	0.867	2.605	0.929	2.169	3.761
		Chemical characteristic band	0.889	2.436	0.932	2.009	3.832
RF	MWS-FD	Full band	0.971	1.199	0.941	2.003	4.111
		CARS	0.974	1.143	0.951	1.814	4.498
		Chemical characteristic band	0.974	1.175	0.941	1.866	4.127
LightGBM	MWS-SNV	Full band	0.977	1.112	0.926	2.035	3.688
		CARS	0.928	1.930	0.796	3.270	2.212
		Chemical characteristic band	0.971	1.207	0.881	2.392	2.897
CNN	MWS-FD	Full band	0.972	1.194	0.946	1.915	4.301
		CARS	0.914	2.097	0.935	2.159	3.777
		Chemical characteristic band	0.929	1.955	0.934	1.990	3.870

**Table 7:** Effects of different wavelength screening methods on the performance of TKS roots powder sample prediction model

Modeling method	Optimal spectral processing	Wavelength screening method	Training set		Validation set		RPD_P_
			${\text{R}}_{{\text{c}}}^{2}$	RMSEC	${\text{R}}_{{\text{P}}}^{2}$	RMSEP	
PLS	MWS-SNV	Full band	0.959	1.524	0.964	1.425	5.307
		CARS	0.951	1.531	0.960	1.429	5.019
		Chemical characteristic band	0.954	1.643	0.975	1.122	6.342
RF	MWS-FD	Full band	0.989	0.781	0.966	1.484	5.461
		CARS	0.988	0.816	0.970	1.361	5.814
		Chemical characteristic band	0.988	0.809	0.979	1.133	6.845
LightGBM	MWS-SNV	Full band	0.985	0.910	0.964	1.427	5.297
		CARS	0.956	1.597	0.923	1.988	3.608
		Chemical characteristic band	0.980	1.091	0.932	1.855	3.835
CNN	MWS-FD	Full band	0.989	0.798	0.970	1.417	5.717
		CARS	0.939	1.872	0.971	1.435	5.515
		Chemical characteristic band	0.980	1.072	0.974	1.280	6.054

#### Evaluation of prediction performance of different models with high NR content intervals

Although the evaluation parameters of the LightGBM prediction model for TKS root segments and powder samples are not particularly impressive compared to other models, the scatter plots of predicted and measured NR content values (Figs. 10, 11) demonstrate that the validation set of this model has superior predictive performance in the high content range (NR content > 15%). As TKS breeding necessitates the selection of individuals with higher NR content from a large number of plants. As shown in Table 8, the RMSEP of the LightGBM model for the root segment samples in the range of high NR content was calculated to be 0.752, which is lower than the RMSEP of PLS, RF, and CNN, all of which are greater than 2. Similarly, the RMSEP of the LightGBM model for root powder sample was 0.918, which is the lowest prediction root mean square error in the high NR content range among the four models. Consequently, in practical breeding work, the collaboration of multiple models is more beneficial for screening samples with high NR content.

**Table 8:** RMSEP statistical table of optimal modeling methods for different models with high gum content (NR content > 15%)

Sample category	Modeling method	Optimal spectral processing	Optimal wavelength screening method	RMSEP
Root segment	PLS	MWS–FD	Chemical characteristic band	2.087
	RF	MWS–FD	CARS	2.022
	LightGBM	MWS–MSC	Full band	0.752
	CNN	MWS–FD	Full band	2.289
Root powder	PLS	MWS–SNV	Chemical characteristic band	1.496
	RF	MWS–FD	Chemical characteristic band	1.170
	LightGBM	MWS–SNV	Full band	0.918
	CNN	MWS–FD	Chemical characteristic band	1.577

## Discussion

The RF model demonstrates the best performance, when Comparing the prediction performance of the entire spectrum interval for root segment and powder models using RPD_P_ as the evaluation criterion. In the Comparison to the widely utilized linear model PLS in the establishment of NR content prediction models used by previous researchers, RF represents superior prediction performance, this suggests that RF may be more suitable for establishing NR content models in TKS. For both root segment and powder models, the most effective spectral preprocessing method is found to be MWS-FD. The main difference between FD and MSC/SNV lies in FD’s FD’s more proficient augmentation of spectral characteristics. Upon the application of the FD, the spectral information related to NR content is significantly enhanced, and the model’s prediction performance was improved. This aligns with the findings of Luo [24]. The performance of the three types of wavelengths (full wavelength, CARS-screened wavelengths, and characteristic wavelengths identified by previous researchers) varies among different models, primarily due to significant differences in model algorithms. Different model structures are suited to different wavelength selection methods, and selecting the most suitable band screening method for the model in practical applications can maximize the model’s effectiveness. The study finds that the prediction performance of the powder sample model is superior to that of the un-milled root segment samples. This is mainly because the rough surface and uneven size control of the un-milled samples lead to much higher environmental errors in spectral acquisition, resulting in poor model performance. Taurines et al. [23] also observed the same phenomenon when establishing the NR prediction model for *P. hysterophorus* L. When comparing the prediction performance of the high content interval (NR > 15%) between root segment and powder models using RMSE as the evaluation criterion, LightGBM’s RMSE is lower than that of the other models, but its prediction performance across the entire interval is not ideal. Therefore, future research can focus on integrating multiple types of models. Currently, commonly used ensemble learning algorithms include Stacking, proposed by Wolpert [52]. This ensemble strategy is a heterogeneous serial learner that integrates various different types of models into an overall system, leveraging the strengths of each model. Employing this algorithm in future research may further optimize the prediction performance of NR content models.

Currently, the majority of near-infrared spectroscopy-based NR content prediction models have predominantly focused on *P. hysterophorus* L. as the subject of study [22–25]. Notably, Chen et al. [26] have contributed to the domain by generating a predictive model for NR content in TKS. Chen et al. utilizing fresh roots of TKS, which encompassed a rubber content ranging from 0.21% to 13.82%, they acquired spectral data via a portable spectrometer and developed a PLS prediction model. The model exhibited the ${\text{R}}_{{\text{P}}}^{2}$ value of 0.97 and the RPD_P_ of 5.90. When compared against the RPD_P_ criterion, the prediction efficacy of the root segment model established in this study appears inferior to that of the fresh root model proposed by Chen et al. Conversely, the powder sample model demonstrated a relatively superior prediction capability. Considering the divergent methodologies employed in the actual measurement of NR content and model development, it would be premature to deduce the superiority of fresh roots or dry roots for the precise determination of NR content. Nonetheless, both quantitative models boast RPDP values significantly exceeding 3, categorizing them as outstanding predictive tools and rendering them suitable for the demands of TKS breeding endeavors. Furthermore, the two distinct near-infrared detection methodologies can serve as complementary approaches. The NR content prediction in fresh roots is apt for on-site rapid assessments, whereas dry roots and powder samples mitigate spectral data distortions caused by inconsistent moisture levels and other variables, making them more appropriate for large-scale screenings where the freshness of samples is not guaranteed. The adoption of diverse near-infrared spectroscopy detection methods stands to facilitate advancements in the TKS industry and breeding activities.

## Conclusion

This article investigates the NR content of TKS dry roots of different growth times by detecting their NR content and collecting spectral data of their dry root segments and powder states. Four spectral preprocessing methods and four modeling methods are compared, and the optimal models for predicting the NR content of TKS root segments and powder were identified as MWS-FD-RF and MWS-FD-CNN respectively. Additionally, the best wavelength selection for each model was determined by comparing the full band, CARS algorithm, and the chemical characteristic wavelengths of NR in TKS. The MWS-FD-CASR-RF model was found to have an ${\text{R}}_{{\text{P}}}^{2}$, RMSEP, and RPD_P_ of 0.951, 1.814, and 4.498 respectively for the root segment sample, while the MWS-FD chemical characteristic band RF model had an ${\text{R}}_{{\text{P}}}^{2}$, RMSEP, and RPD_P_ of 0.979, 1.133, and 6.845 respectively for the powder sample. The RPD_P_ of both models was greater than 3, indicating excellent predictive performance. The powder sample had higher ${\text{R}}_{{\text{P}}}^{2}$, RPD_P_, and lower RMSE compared to the root segment sample, indicating better performance of the powder prediction model. Furthermore, the RMSEP of the LightGBM model for TKS root segments and powder samples reached 0.752 and 0.918 respectively in the range of more than 15% NR content, suggesting that combining multiple models is likely to be more suitable for practical applications.

## Data Availability

Please contact the corresponding author for data requests.
